# Partial depletion and repopulation of microglia have different effects in the acute MPTP mouse model of Parkinson’s disease

**DOI:** 10.1111/cpr.13094

**Published:** 2021-07-26

**Authors:** Qing Li, Chenye Shen, Zhaolin Liu, Yuanyuan Ma, Jinghui Wang, Hongtian Dong, Xiaoshuang Zhang, Zishan Wang, Mei Yu, Lei Ci, Ruilin Sun, Ruling Shen, Jian Fei, Fang Huang

**Affiliations:** ^1^ Department of Translational Neuroscience State Key Laboratory of Medical Neurobiology MOE Frontiers Center for Brain Science Jing' an District Centre Hospital of Shanghai Institutes of Brain Science Fudan University Shanghai China; ^2^ Shanghai Engineering Research Center for Model Organisms Shanghai Model Organisms Center INC Shanghai China; ^3^ Joint Laboratory for Technology of Model Organism Shanghai Laboratory Animal Research Center and School of Life Science and Technology Tongji University; ^4^ Shanghai Laboratory Animal Research Center Shanghai China; ^5^ School of Life Science and Technology Tongji University Shanghai China

**Keywords:** microglial depletion, microglial repopulation, nigrostriatal axis, Parkinson's disease

## Abstract

**Objectives:**

Parkinson's disease (PD) is a common neurodegenerative disorder characterized by the progressive and selective degeneration of dopaminergic neurons. Microglial activation and neuroinflammation are associated with the pathogenesis of PD. However, the relationship between microglial activation and PD pathology remains to be explored.

**Materials and Methods:**

An acute regimen of MPTP was administered to adult C57BL/6J mice with normal, much reduced or repopulated microglial population. Damages of the dopaminergic system were comprehensively assessed. Inflammation‐related factors were assessed by quantitative PCR and Multiplex immunoassay. Behavioural tests were carried out to evaluate the motor deficits in MPTP‐challenged mice.

**Results:**

The receptor for colony‐stimulating factor 1 inhibitor PLX3397 could effectively deplete microglia in the nigrostriatal pathway of mice via feeding a PLX3397‐formulated diet for 21 days. Microglial depletion downregulated both pro‐inflammatory and anti‐inflammatory molecule expression at baseline and after MPTP administration. At 1d post‐MPTP injection, dopaminergic neurons showed a significant reduction in PLX3397‐fed mice, but not in control diet (CD)‐fed mice. However, partial microglial depletion in mice exerted little effect on MPTP‐induced dopaminergic injuries compared with CD mice at later time points. Interestingly, microglial repopulation brought about apparent resistance to MPTP intoxication.

**Conclusions:**

Microglia can inhibit PD development at a very early stage; partial microglial depletion has little effect in terms of the whole process of the disease; and microglial replenishment elicits neuroprotection in PD mice.

## Graphical abstract

PLX3397 can efficiently eliminate microglia in the nigrostriatal pathway; Microglia are neuroprotective at early time post‐MPTP injection; Microglial depletion has little effect on the progression of PD in mice; Repopulated microglia exhibit a protective effect in MPTP‐induced PD mice.Microglial depletion in the nigrostriatal pathway has little effect on the progression of PD in mice, while repopulated microglia exhibit a protective effect in MPTP mouse models.

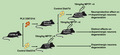

## 1 INTRODUCTION

Parkinson's disease (PD) is the second most common neurodegenerative disease, threatening human health. PD is clinically characterized by symptoms such as resting tremor, bradykinesia, rigidity and postural instability, accompanying non‐motor symptoms such as olfactory hypotension, sleep disturbance, depression and anxiety.^1^, ^2^, ^3^ The main pathological features of PD are the progressive loss of dopaminergic neurons and the deposition of Lewy bodies (LB).^4^, ^5^, ^6^ Microglia, as the resident immune cells of the central nervous system (CNS) and of high heterogeneity, play critical roles in CNS homeostasis and functions. Under different stimulation, they can polarize into pro‐inflammatory M1 microglia and anti‐inflammatory M2 microglia, in which conditions TNFα, IL1β, IL6, CD16 and CD86 or TGFβ, IL4, IL10, CD206, CCL22 and TREM2 are upregulated, respectively.^7^ In the conditions of brain injury and neurodegenerative diseases, both neuroprotective and detrimental effects of microglia have been revealed. The development of pharmacological inhibition and genetic targeting (eg, DTR expressing exclusively in microglia), which can deplete microglial cells or suppress microglial activation in vivo by specific inhibitors or DT toxin, has much expanded our understandings of microglia.^8^ Microglial activation and neuroinflammation are common features in the early stage of Parkinson's disease, suggesting that neuroinflammation contributes to the onset and progression of PD.^9^, ^10^ The progressive death of dopaminergic neurons in PD causes the release of endogenous damage‐associated molecular patterns (DAMP), which leads to excessive activation of microglia, and subsequently, activated microglia elicit an inflammatory response.^11^, ^12^, ^13^ Maintenance of the microglial population depends on the continuing activation of the receptor for colony‐stimulating factor 1 (CSF1R), which is also essential for microglial development.^14^, ^15^, ^16^ The pharmacological blockade of CSF1R by PLX3397 and many other similar inhibitors lead to a rapid depletion of microglia, and microglia reach full repopulation soon after removal of the inhibitors.^14^, ^17^, ^18^, ^19^, ^20^, ^21^ With the strategy of microglial elimination, Yang et al showed that microglial ablation led to the augmented dopaminergic neurotoxicity in MPTP‐treated mice,^18^ while Oh et al found that in 6‐OHDA‐induced PD rats, depletion of microglia elicited beneficial effects on motor and non‐motor symptoms of PD.^22^ Moreover, in rotenone‐induced mouse PD models, microglial activation contributed to neurodegeneration in the locus coeruleus and cognitive impairments.^23^, ^24^ Thus, whether microglia are beneficial or harmful remains controversial in PD. And the effects of repopulated microglia in PD are also warranted to illustrate.

In this study, to clarify the function of microglia in Parkinson's disease, an acute regimen of MPTP was administered to adult C57BL/6J mice with a normal, much reduced or repopulated microglial population. We comprehensively analysed the impact of microglia at different stages in PD animal models, including the damages of the nigrostriatal pathway, the levels of inflammation, the changes in glial cells and the mouse behaviours. Our results demonstrate that microglial depletion has little effect on dopaminergic injuries; however, repopulated microglia elicits neuroprotection, in PD mice.

## 2 MATERIALS AND METHODS

### 2.1 Animal research

Male C57BL/6J mice, 10 weeks old and weighing 26‐29 g, were obtained from the Shanghai Model Organisms Center, Inc All animal experiments were conducted according to the guideline of the Institutional Animal Care and Use Committee of Fudan University, Shanghai Medical College. All efforts were made to minimize adverse effects.

### 2.2 Compounds

PLX3397 (Pexidartinib; Selleckchem, USA) was formulated in AIN‐76A standard chow (FBSH Biopharmaceutical Co., Ltd., Shanghai, China) at a concentration of 290 mg/kg. Normal AIN‐76A diet was served as the control.

Mice were fed with a PLX3397‐formulated diet (PLX3397) for 7, 10, 14 and 21 days to determine the optimal days for microglial elimination and repopulation in the nigrostriatal pathway (n = 3 per group). After 21 days, a subset of mice with the PLX3397 diet were switched to a control diet (CD) for 4 and 7 days (n = 3 per group) to repopulate microglia.

Mice were fed with a PLX3397 diet or a control diet for 21 days and followed by a diet according to the requirements of different experiments to clarify the function of microglia in PD (n = 4‐5 per group). The design of each experiment was shown as a diagram, respectively.

### 2.3 Mouse treatment

Mice were intraperitoneally injected with MPTP∙HCl at a dose of 10 mg/kg (Sigma, USA) or normal saline (NS) for four times at 2 h of intervals. Animals were sacrificed at different time points after the last injection.

### 2.4 Immunofluorescence

Animals were anaesthetized and perfused transcardially with normal cold saline, followed by 4% paraformaldehyde. Brains were collected and post‐fixed with 4% paraformaldehyde overnight and subsequently immersed in 15% and 30% sucrose at 4°C overnight. Dissected brains were embedded in the OCT compound then cut into coronal sections (30 μm) by a freezing microtome and stored in a cryoprotectant solution at −20°C for further analysis. The sections were rinsed and permeabilized with PBS containing .5% Triton X‐100 for 20 min, blocked in 10% normal donkey serum containing 0.05% Tween‐20 for .5 h and then incubated with primary antibodies at 4°C overnight. The following antibodies were used as follows: mouse anti‐TH (1:1000, ImmunoStar, USA), chicken anti‐GFAP (1:500, EnCor Biotechnology, USA) and rabbit anti‐Iba1 (1:500, Abcam, USA). Appropriate secondary antibodies conjugated with Alexa fluorophore 488, 594 or 647 were used for visualization. 6‐Diamidino‐2‐phenylindole (DAPI) was used for nuclear counterstaining. Images were captured by a Zeiss LSM 800 confocal microscope (Germany).

### 2.5 Western blot

Protein samples were lysed in RIPA lysed buffer containing protease inhibitor cocktail (1:100, BioMake, China). The lysates were centrifuged at 16 000g for 15 min at 4°C, and then, the supernatants were collected. The protein concentration of samples was determined using a BCA protein assay kit (Beyotime, China). Equal amounts of 30 µg total proteins were loaded onto SDS‐PAGE. Following electrophoresis, proteins were transferred to Immobilon‐PSQ membranes (Merck Millipore, USA). Blots were probed with mouse anti‐TH (1:2000, ImmunoStar, USA) and mouse anti‐GFAP (1:2000, Merck Millipore, USA) at 4°C overnight. After washing, the membranes were incubated with appropriate secondary antibodies for 2 h and detected by an Odyssey infrared imaging system (LI‐COR, USA).

### 2.6 Quantitative real‐time PCR assays

The total RNA was extracted using TRIzol reagent according to the manufacturer's protocols. Quantitative analysis of RNA was performed by using NanoDrop spectrophotometer. 2 µg total RNA was used for complementary DNA (cDNA) synthesis using the One‐Step gDNA Removal and cDNA Synthesis SuperMix (Transen, China). Real‐time PCR was performed in duplicates with a quantitative thermal cycler (Thermo Fisher Scientific, USA). The following PCR conditions were used: 95°C for 15 s, 62°C for 20 s and 72°C for 20 s for 40 cycles (with a final reaction volume of 20 µl). A comparative threshold cycle method was used to quantify target mRNAs, and all samples were normalized to GAPDH (as the internal reference gene) using the 2^‐ΔΔCt^ method. Fold changes for each treatment were normalized and are shown as percentages of the control. The primers used in real‐time PCR were as follows:

*C1q*, 5’‐AGCATCCAGTTTGATCGGAC‐3’ and 5’‐CTTCAGCCACTGTCCATACTAG‐3’; *COX2*, 5’‐GAGCAACTATTCCAAACCAGC‐3’ and 5’‐AGCTCTGGGTCAAACTTGAG‐3’; *iNOS*, 5’‐TGGAGCGAGTTGTGGATTG‐3’ and 5’‐CGTAATGTCCAGGAAGTAGGTG‐3’; *TNFα*, 5’‐CTATGTCTCAGCCTCTTCTCATTC‐3’ and 5’‐TGGGAACTTCTCATCCCTTTG‐3’; *NLRP3*, 5’‐ATGGGTTTGCTGGGATATCTC‐3’ and 5’‐GCGTTCCTGTCCTTGATAGAG‐3’; *ASC*, 5’‐TGCTTAGAGACATGGGCTTAC‐3’ and 5’‐CAATGAGTGCTTGCCTGTG‐3’; *IL18*, 5’‐CTTCGTTGACAAAAGACAGCC‐3’ and 5’‐CACAGCCAGTCCTCTTACTTC‐3’; *IL1α*, 5’‐TTCTGCCATTGACCATCTCTC‐3’ and 5’‐GTTGCTTGACGTTGCTGATAC‐3’; *IL1β*, 5’‐CTACCTGTGTCTTTCCCGTG‐3’ and 5’‐TGCAGTTGTCTAATGGGAACG‐3’; *IL6*, 5’‐CTTCACAAGTCGGAGGCTTAAT‐3’ and 5’‐CAGTTTGGTAGCATCCATCATTTC‐3’; *Caspase1*, 5’‐AATGTGTCTTGGAGACATCCTG‐3’ and 5’‐GTGGGCATCTGTAGCCTAAAT‐3’; *GAPDH*, 5’‐GCCTTCCGTGTTCCTACC‐3’ and 5’‐CCTCAGTGTAGCCCAAGATG‐3’; *Iba1*, 5’‐CGATGATCCCAAATACAGCAATG‐3’ and 5’‐CCCAAGTTTCTCCAGCATTC‐3’; *GFAP*, 5’‐GAAAACCGCATCACCATTCC‐3’ and 5’‐CTTAATGACCTCACCATCCCG‐3’; *IFN‐γ*, 5’‐ATCAGGCCATCAGCAACAA‐3’ and 5’‐ACCTGTGGGTTGTTGACCTC‐3’; *MHCⅠ*, 5’‐GTCCTTCAGCAAGGACTGGTC‐3’ and 5’‐TGGATTTGTAATTAAGCAGGTTC‐3’; *MHCⅡ* 5’‐ACACGGTGTGCAGACACAA‐3’ and 5’‐TCAGGCTGGGATGCTCC‐3’; *CD206*, 5’‐CAAGGAAGGTTGGCATTTGT‐3’ and 5’‐CCTTTCAGTCCTTTGCAAGC‐3’; YM1/2, 5’‐CAGGGTAATGAGTGGGTTGG‐3’ and 5’‐CACGGCACCTCCTAAATTGT‐3’; *Arginase‐1*, 5’‐AGCCAATGAAGAGCTGGCTGGT‐3’ and 5’‐AACTGCCAGACTGTGGTCTCCA‐3’; *BDNF*, 5’‐CTGAGCGTGTGTGACAGTATTA‐3’ and 5’‐CTTTGGATACCGGGACTTTCTC‐3’; *GDNF*, 5’‐TGAAGACCACTCCCTCGG‐3’ and 5’‐GCTTGTTTATCTGGTGACCTTTTC‐3’; *NGF*, 5’‐CCCAATAAAGGTTTTGCCAAGG‐3’ and 5’‐TTGCTATCTGTGTACGGTTCTG‐3’; *EGF*, 5’‐AACGCCGAAGACTTATCCAG‐3’ and 5’‐TGAAGACGTACCCTGTTTTGG‐3’; *FGF1*, 5’‐TGGGACAAGGGACAGGAG‐3’ and 5’‐TCCTCATTTGGTGTCTGCG‐3’; *TGFβ1*, 5’‐CCTGAGTGGCTGTCTTTTGA‐3’ and 5’‐CGTGGAGTTTGTTATCTTTGCTG‐3’; *IGF1*, 5’‐TGGATGCTCTTCAGTTCGTG‐3’ and 5’‐AGTACATCTCCAGTCTCCTCAG‐3’; *TREM2*, 5’‐ACAGCACCTCCAGGAATCAAG‐3’ and 5’‐AACTTGCTCAGGAGAACGCA‐3’; *CD11b*, 5’‐CCTTCATCAACACAACCAGAGTGG −3’ and 5’‐CGAGGTGCTCCTAAAACCAAGC‐3’.

### 2.7 Multiplex immunoassay

Protein samples were lysed in a T‐PER lysed buffer containing protease inhibitor cocktail (1:100, Biomake, China). The lysates were centrifuged at 16,000g for 15 min at 4°C, and then, the supernatants were collected. Protein concentration was determined using a BCA protein assay kit (Thermo Fisher Scientific, USA). According to the manufacturer's protocols, the Luminex Mouse Magnetic Assay kit (R&D Systems, USA) was used to measure cytokines.

### 2.8 High‐performance liquid chromatography

The dissected striatum was homogenized in .4 M HClO_4_ for 30 s. The lysates were centrifuged at 12,000g for 10 min at 4°C. Then, the supernatants were collected to determine the concentrations of DA and its metabolites homovanillic acid (HVA) and 3, 4‐dihydroxyphenylacetic acid (DOPAC), as well as serotonin (5‐HT) and hydroxyindole acetic acid (5‐HIAA) by using the chromatograph (ESA, USA) with a 5014B electrochemical detector.

### 2.9 Animal behaviour studies

#### 2.9.1 Rotarod test

One day before the experiment, mice were pre‐trained on a rotarod instrument (MED Associates, USA) three times, separated by one‐hour intervals. At testing days, mice were placed on the rod rotating at a speed of 20 rpm, and the latency to fall and times of drop were measured. Total time spent on the rotarod was measured up to a maximum of 300 s.

#### 2.9.2 Pole test

The pole test was carried out 3 and 7 days after MPTP injection to evaluate the motor function impairment. A wood pole with a rough surface, 75cm in length and 1cm in diameter, was placed vertically in the cage of mice to be tested. Briefly, mice were placed with their head facing upside on top of the pole; the time required for the mice to turn around and climb down was recorded to assess the movement initiation and coordination.

#### 2.9.3 Cell counting

Stereological cell counting was conducted to measure the density of TH‐positive cells in the substantia nigra par compacta (SNpc) using a Stereo Investigator system (Micro Brightfield, USA) attached to a Leica microscope as previously described^25^ (in Figures 2C, 6C and Figure S5C). One out of five 30 μm‐thick sections and a total of 6 sections from Bregma −2.80 to −3.80 mm were collected and analysed. The substantia nigra was delineated using a 4×objective, and the actual counting was performed under a 40×objective. Assays were performed in a double‐blind fashion by two operators.

**FIGURE 1:**
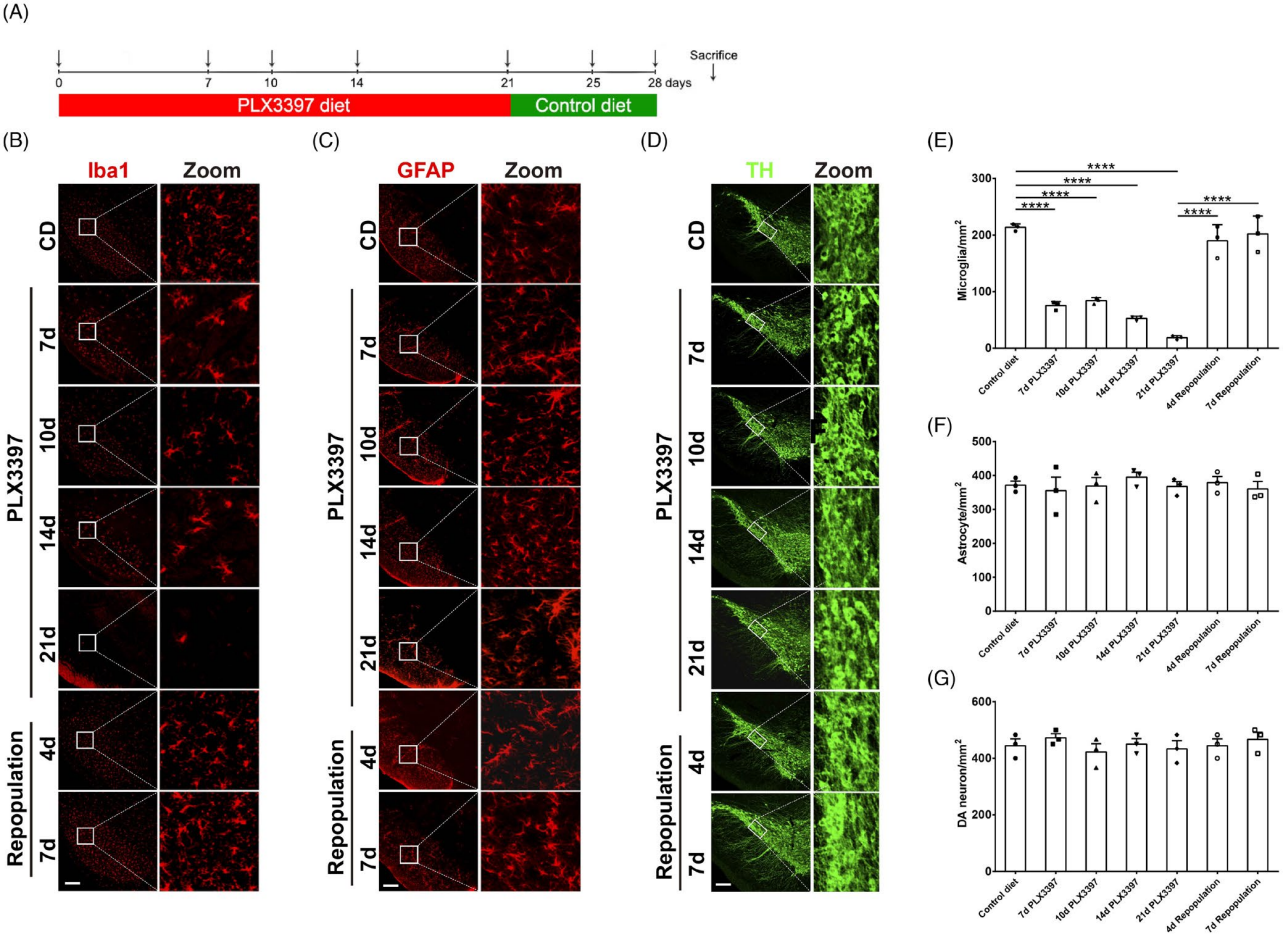
The effect of microglial elimination and repopulation with the specific CSF1R inhibitor PLX3397 in the mouse substantia nigra. (A) Schematic schedule of microglial depletion and repopulation models with CSF1R inhibitors PLX3397. (B, E) Representative Iba1 (red)immunofluorescence staining (A) and quantification of Iba1^+^ microglial cells (E) the substantia nigra during the treatment of PLX3397 and after the repopulation of microglia. (C, F) Representative GFAP (red) immunofluorescence staining (C) and quantification of GFAP^+^ astrocytic cells (F) in the substantia nigra during the treatment of PLX3397 and after the repopulation of microglia. (D, G) Representative TH (green) immunofluorescence staining (D) and quantification of TH^+^ cells (G) in the substantia nigra during the treatment of PLX3397 and after the repopulation of microglia. CD: control diet; PLX3397: PLX3397‐formulated diet. One‐way ANOVA followed by Holm‐Sidak’s multiple comparisons test was used for statistical analysis. ^****^ *P*<.0001. n=3

**FIGURE 2:**
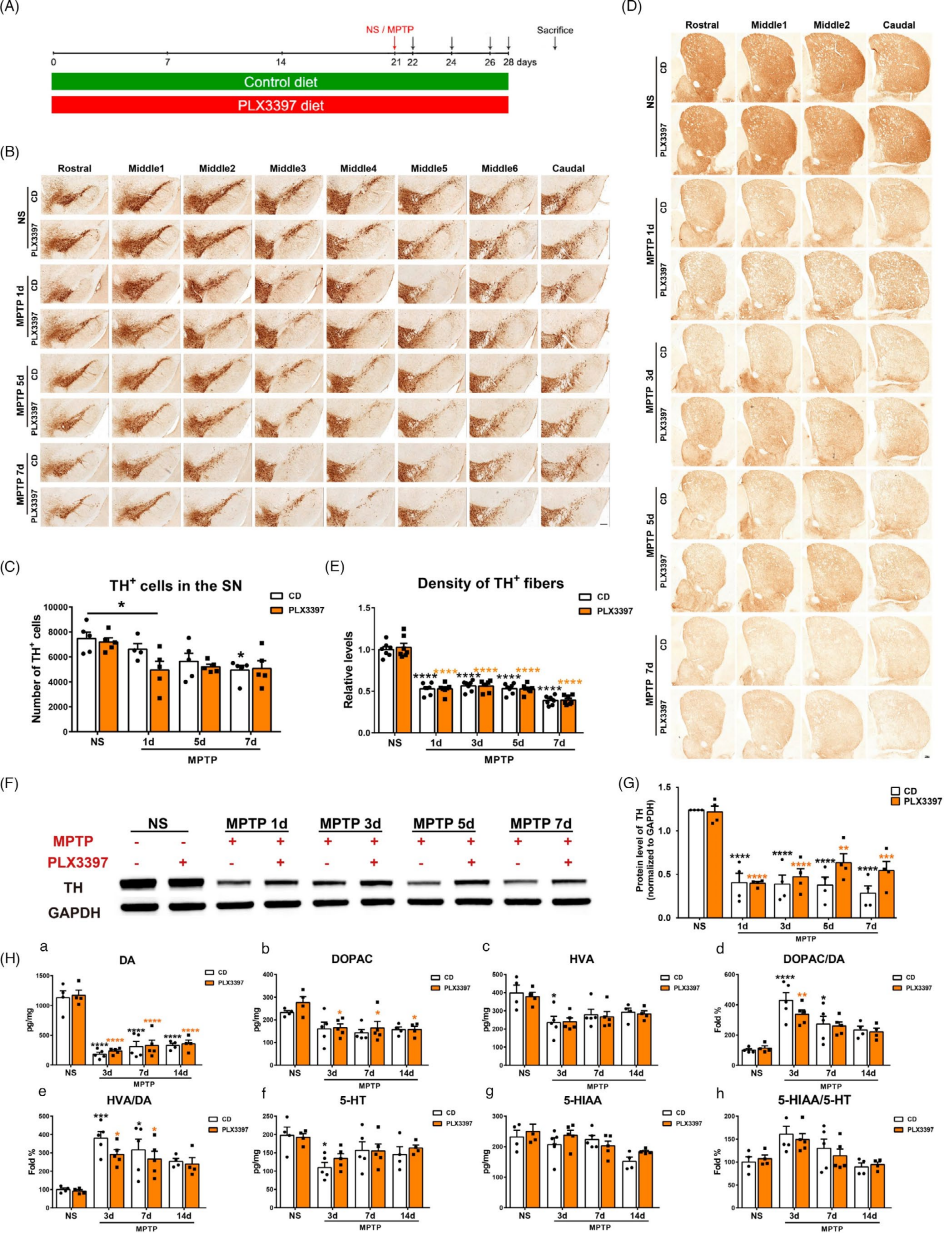
The effect of partial microglial depletion on dopaminergic degeneration in the nigrostriatal pathway after MPTP administration. (A) Schematic schedule of the MPTP‐driven PD mice models. (B, C) Immunohistochemical staining (B) and stereological count of TH^+^ cells (C) in the SNpc of NS‐ treated mice and MPTP‐treated mice at 1d, 5d, 7d post‐injection. Scale bar: 200 μm. n=4‐5. (D, E) Immunohistochemical staining showing TH^+^ fibers. Scale bar: 200 μm (D) and quantification of TH^+^ fiber density (E) in the striatum. n=7. (F, G) Western blot analysis of striatal TH protein levels, GAPDH served as the control (F) and quantification of TH protein levels (G). n=4. (H) HPLC assays of the striatal dopamine (A) and its metabolites DOPAC (b) and HVA (c), 5‐HT (f) and its metabolite 5‐HIAA (g) after MPTPadministration. Ratios of DOPAC to DA (D),HVA to DA (E), and 5‐HIAA to 5‐HT (H) were also shown. n=4‐5. NS: normalsaline; CD: control diet; PLX3397: PLX3397‐formulated diet. Two‐way ANOVA followed by Holm‐Sidak’s multiple comparisons test was used for statistical analysis. ^*^ *P*<.05,^**^ *P*<.01, ^***^ *P*<.001, ^****^ *P*<.0001, vs respective NSgroups, except in (C), ^*^ *P*<.05, vs CD‐NS group

**FIGURE 3:**
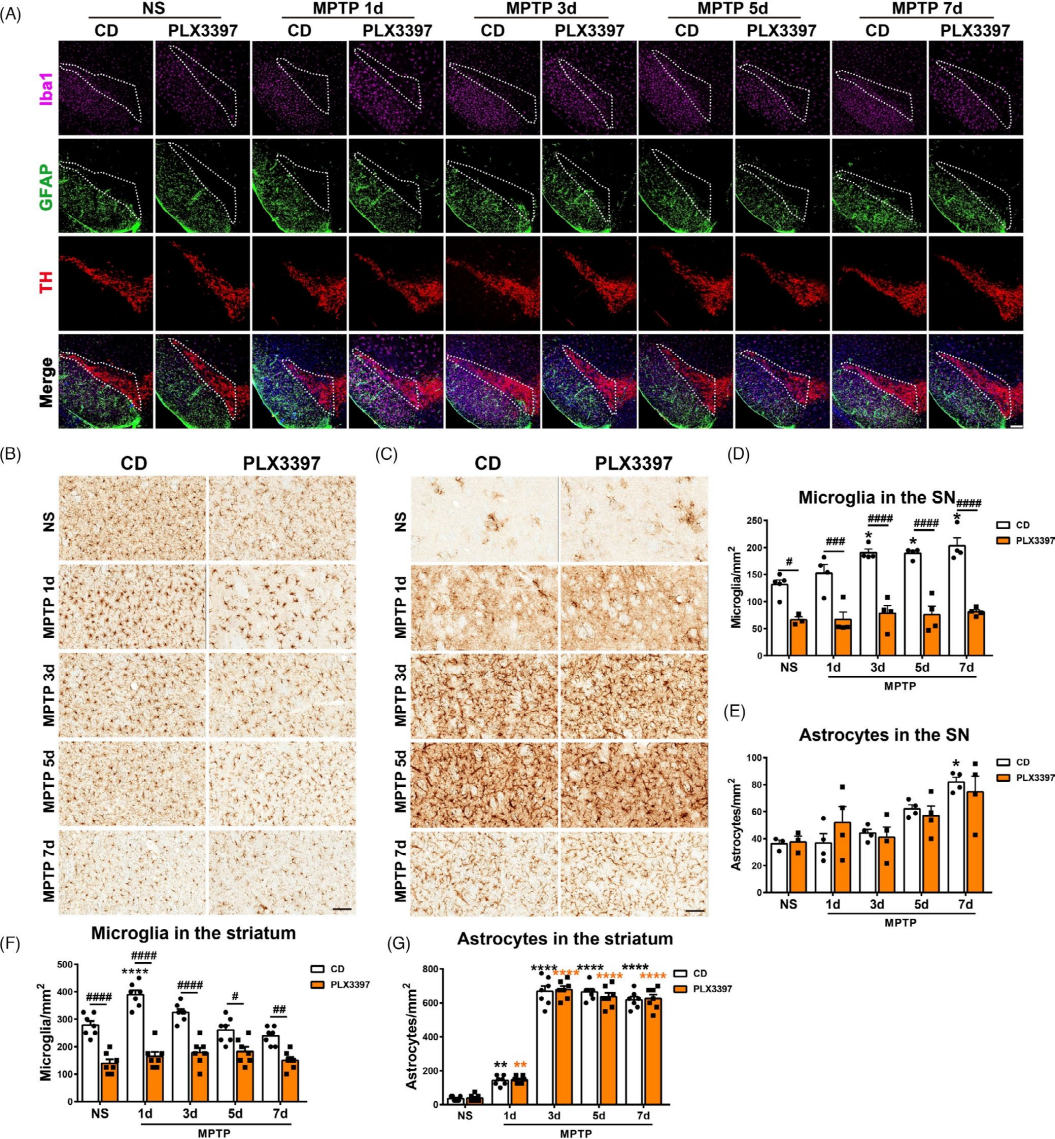
Analysis of microgliaand astrocytes in nigrostriatal pathway of mice with partial microglialdepletion after MPTP administration. (A) Immunofluorescencestaining of Iba1^+^ (pink), GFAP^+^ (green) and TH^+^(red) in the SNpc. Scale bars: 200 μm. (B,C) Immunohistochemical staining showing Iba1^+^ (B) and GFAP^+^ (C) cells in the striatum.Scale bars: 100 μm. (D, E) Quantification of Iba1^+^ microglialcells (D) and GFAP^+^ astrocyticcells (E) in the SNpc. n=3‐6. (F, G) Quantification of Iba1^+^ microglial cells (F) and GFAP^+^ astrocytic cells (G) in the striatum. n=7. NS: normal saline; CD: control diet;PLX3397: PLX3397‐formulated diet. Two‐way ANOVA followed by Holm‐Sidak’smultiple comparisons test was used for statistical analysis. ^**^ *P*<.01, ^***^ *P*<.001, ^****^ *P*<.0001, vs respective NS groups. ^#^ *P*<0.05, ^##^ *P*<.01, ^###^ *P*<.001, ^####^ *P*<.0001, CD groups vs PLX3397 groups

The analysis of dopaminergic cells, microglia and astrocytes was carried out by counting the number of positive cells (in Figure 1E, F, G). For mouse substantia nigra, the slices with the largest number of TH‐positive cells (Bregma −2.92 to −3.08) were chosen. Quantitation of TH‐positive cells was accomplished by using an object area to counting the number. To count the microglia and astrocytes in the substantia nigra par compacta (SNpc) (in Figures 3D, E and 6F, G), an image‐based counting approach was adopted in this paper.^26^ Briefly, the regions of the SNpc on each section were depicted by dotted line based on TH immunostaining. Then, the positive immunofluorescent signals of Iba1 or GFAP were counted, and the actual area of the SNpc was measured by ImageJ software (NIH Image).

**FIGURE 4:**
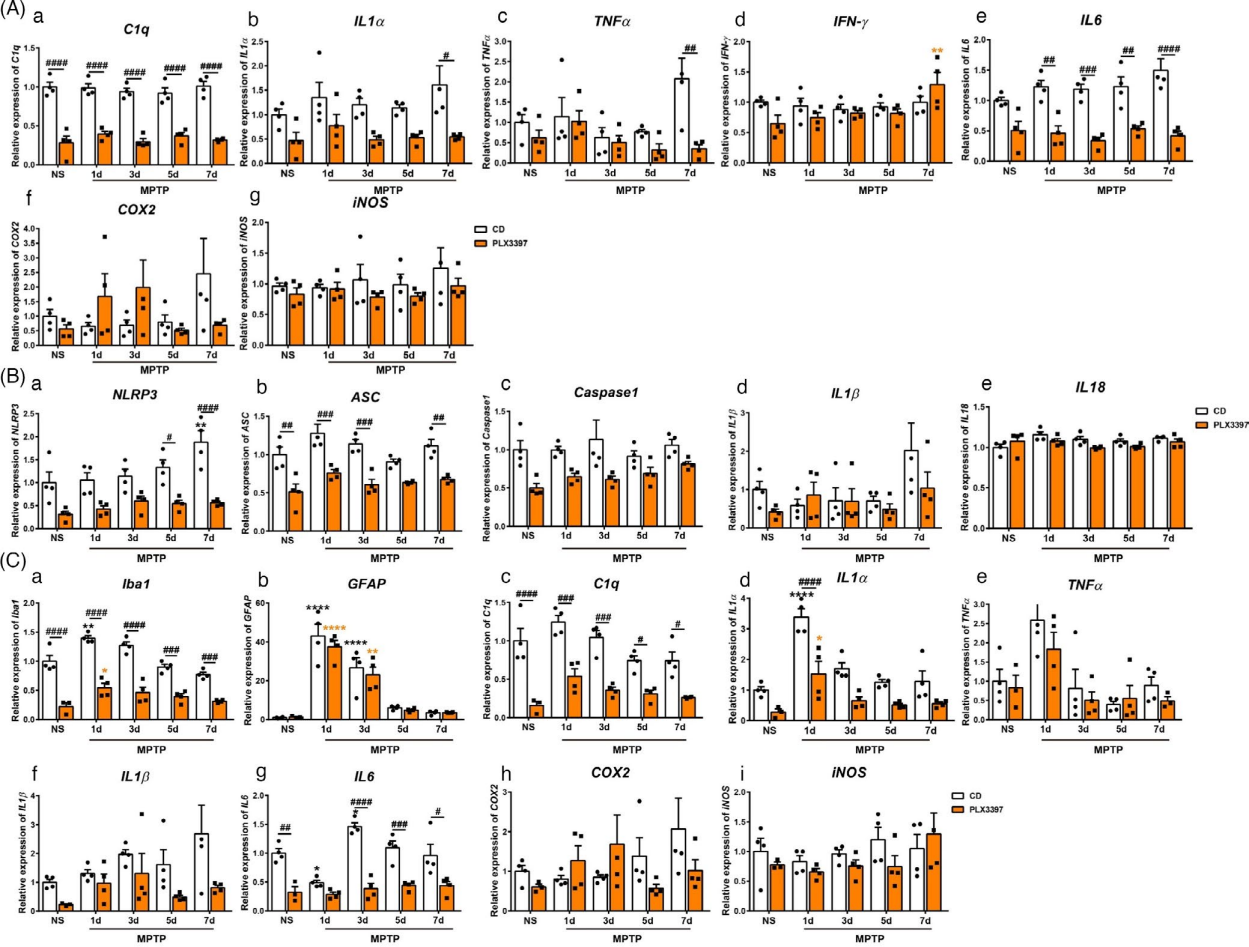
The transcripts of inflammation‐related molecules in the nigrostriatal pathway of mice after MPTP administration. (A) The transcripts of *C1q* (a), *IL1α*(b), *TNFα* (c), *IFN‐γ* (d), *IL6* (e), *COX2* (f) and *iNOS* (g) in the SN. (B) The transcripts of *NLRP3* (a), *ASC* (b), *Caspase1* (c), *IL1β* (d) and *IL18* (e) in the SN. (C) The transcripts of *Iba1* (a), *GFAP* (b), *C1q* (c), *IL1α* (d), *TNFα* (e), *IL1β* (f), *IL6* (g), *COX2*(h) and *iNOS* (i) in the striatum. NS: normal saline. CD: control diet; PLX3397: PLX3397‐formulated diet. *GAPDH* served as the reference gene. Two‐way ANOVA followed by Holm‐Sidak’s multiple comparisons test was used for statistical analysis. ^*^ *P* < .05, ^**^ *P* < .01, ^****^ *P* < .0001, vs respective NS groups; ^#^ *P* < .05, ^##^ *P* < .01, ^###^ *P* < .001, ^####^ *P* < .0001, CD groups vs PLX3397 groups. n = 3‐4

#### 2.9.4 Statistical analysis

All data were expressed as means ± SEM. Data were assessed for normal distribution by the Shapiro‐Wilk test. Unpaired two‐tailed Student's t tests were used to compare two groups. One‐way or two‐way analysis of variance (ANOVA) followed by Holm‐Sidak's or Dunn's multiple comparisons test was used to compare multiple groups as appropriate. Statistical analysis was performed using the Prism 7 software (GraphPad Software Inc, USA). *P* < .05 was considered statistically significant. The interaction between the treatments was tested, and corresponding *F*‐, dfn‐, dfd‐ and *P*‐values of significant interaction were showed.

## 3 RESULTS

### 3.1 PLX3397 dramatically reduces the microglial population in the substantia nigra, but has no obvious effect on the basal status of nigrostriatal pathway in mice

To evaluate whether microglial depletion affects the nigrostriatal pathway in mice, we first established an appropriate treatment paradigm for the microglial depletion and repopulation models with CSF1R inhibitors PLX3397. C57BL/6 mice were fed with a PLX3397‐formulated diet for up to 21 days (Figure 1A). Mice showed a dramatic reduction in body weight 7 days after PLX3397 treatment, which returned straightly back to normal body weight afterwards (Figure S1A). As determined by microglia‐specific marker Iba1 staining, microglial numbers were substantially reduced to 30% in the substantia nigra (SN) 7 days after the treatment of PLX3397, and continuous suppression of CSF1R for 21 days led to 90% microglial depletion in the SN (Figure 1B, E). In a subset of microglia‐depleted mice, we withdrew the treatment for either 4 or 7 days to allow microglia to repopulate. The SN region was rapidly replenished with repopulated microglia 4 or 7 days after the cessation of PLX3397 diet (Figure 1B, E). The astrocyte population was also evaluated by counting fibrillary acidic protein (GFAP) immunoreactive cells within the SN and showed no alteration during the processes of microglial depletion and repopulation (Figure 1C, F). Furthermore, immunostaining of tyrosine hydroxylase (TH), a dopaminergic neuronal marker, showed that the number of TH^+^ dopaminergic neurons had no significant difference between the treated and the control mice at all time points (Figure 1D, G), indicating there was little effect of microglial depletion on dopaminergic neuronal population in the SN. Moreover, the striatal neurotransmitters and the mouse motor behavior did not alter as well (Figure S1B‐D).

### 3.2 Partial depletion of microglia has distinct effects on dopaminergic neurons degeneration in the processes of MPTP‐induced injuries

An acute regimen of MPTP (10 mg/kg body weight) was adopted in this study. Mice were treated with a control diet (CD) or PLX3397‐formulated diet (PLX3397) for consecutive 21 days before MPTP or NS administration. Treatment was sustained until the end of the experiments (Figure 2A). Therefore, there were four experimental groups: CD‐NS, CD‐MPTP, PLX3397‐NS and PLX3397‐MPTP. First, whether microglial depletion affected the dopaminergic neuronal degeneration was assessed. Immunohistochemistry and stereological cell counting revealed that compared to CD‐NS mice, dopaminergic neurons were significantly reduced in the SNpc of PLX3397‐MPTP mice, but not in CD‐MPTP mice at 1 day post‐injection. The numbers of dopaminergic neurons decreased significantly in CD‐MPTP mice at 7 days after the challenge, and they did not vary between CD‐MPTP mice and PLX3397‐MPTP mice groups (Figure 2B, C). Then, TH protein levels were determined by Western blot, and the reduction in TH protein in the SN reached the maximum at 3 days post‐MPTP injection, with no difference between CD‐ and PLX3397‐treated mice (Figure S2A, B).

In the striatum, immunohistochemistry staining showed that the density of TH‐positive nerve fibres decreased dramatically after MPTP administration, whereas it did not differ between two groups of mice at all experimental time points (Figure 2D, E). Similarly, striatal protein levels of TH were determined by Western blot assays, which were dramatically reduced from 1d up to 7d after MPTP intoxication, and the reduction was close between the two groups of mice (Figure 2F, G). Furthermore, the striatal levels of dopamine (DA), DOPAC, HVA, 5‐HT and 5‐HIAA were analysed by HPLC assay. In striatum of mice challenged with MPTP, the levels of DA and its metabolites DOPAC and HVA decreased, which were comparable between the CD and PLX3397 groups (Figure 2H a‐c); and the levels of neurotransmitter 5‐HT and its metabolite 5‐HIAA did not differ at all experimental time points between the two groups (Figure 2H f, g). In addition, there were no differences in the ratios of DOPAC to DA, HVA to DA and 5‐HIAA to 5‐HT between CD‐ and PLX3397‐treated mice (Figure 2H d, e, h). Thus, depletion of microglia by PLX3397 plays a quite faint role in the dopaminergic neuron degeneration in MPTP‐driven PD mice.

### 3.3 Partial depletion of microglia causes suppressed microglial proliferation and has no effect on the activation of astrocytes in nigrostriatal pathway of MPTP‐driven PD mice

Next, the microglial proliferation and activation of astrocytes were assessed in this model. Through Iba1 immunofluorescence staining and cell counting, we found that the numbers of microglia in the SN of CD‐treated mice reached the peak value approximately at 3 to 7 days after MPTP administration; however, they were significantly reduced and did not proliferate in the SN of PLX3397‐treated mice at all the time points (Figure 3A, D). Notably, the numbers of astrocytes did not differ between the CD and PLX3397 groups, though astrocyte number in the SN of CD‐MPTP mice increased dramatically at 7 days post‐injection (Figure 3A, E).

In the striatum, the numbers of microglia increased rapidly in CD‐treated mice and restored at 5 days after MPTP administration; on the contrary, microglial cells did not proliferate in PLX3397‐treated mice during the whole experimental process [F(4, 60) = 7.388, *P* < .0001; Figure 3B, F]. In terms of astrocytes, we found that MPTP exposure elicited increases in the astrocyte densities at 1 day after MPTP administration and maintained activated afterwards, whereas there was no difference between the two groups (Figure 3C, G). Besides, Western blot assays showed that the striatal GFAP protein levels were upregulated in the striatum at 3 days after MPTP injection, whereas there was no difference between the CD and PLX3397 groups (Figure S3 A, B).

### 3.4 Partial microglial depletion attenuates the inflammatory response in MPTP‐treated mice

Microglia produce a series of neurotrophic factors and inflammation‐related molecules, ultimately supporting neuronal viability or damaged neurons. The activation of microglia in both striatum and substantia nigra has been well documented in MPTP‐treated mice^27^, ^28^, ^29^, ^30^; however, it is not fully clear whether microglial depletion induces different inflammatory responses. Here, we detected the expression of inflammatory factors in the nigrostriatal pathway after MPTP administration. The expression patterns of pro‐inflammatory molecules between the CD and PLX3397 groups manifested complex changes. Compared to CD‐treated mice, *C1q* transcripts were markedly reduced in the substantia nigra of microglial‐depleted mice at all the experimental time points, while *IL6* transcripts were decreased in microglial‐depleted mice after MPTP administration, and the transcripts of *IL1α* and *TNFα* [F(4, 30) = 2.981, *P* = .0348] were markedly decreased in mice with microglial depletion at 7d post‐MPTP injection. Notably, 7 days after MPTP treatment, the *IFN‐γ* transcripts were elevated significantly in microglial‐depleted mice compared with their PLX3397‐NS controls. In addition, the expression of *IFN‐γ*, *COX2* and *iNOS* showed no difference between the CD and PLX3397 groups (Figure 4A). Given that previous studies had reported that the NLRP3 inflammasome was activated in the substantia nigra of MPTP‐challenged mice,^28^, ^31^, ^32^, ^33^ we further evaluated whether microglial depletion affected the activation of the NLRP3 inflammasome. As shown, microglial depletion significantly reduced the expression of inflammasome‐relevant molecules in the SN, including *NLRP3* and *apoptosis‐associated speck‐like protein* (*ASC*), and *NLRP3* transcript increased significantly in CD‐MPTP mice at 7 days post‐injection compared with their CD‐NS controls (Figure 4B), whereas the transcripts of *Caspase1*, *IL1β* and *IL18* did not alter between the CD and PLX3397 groups (Figure 4B). Intriguingly, a remarkable increase in *IL18* transcript was detected in the CD‐MPTP mice at 14 days post‐injection, but not in PLX3397‐MPTP mice (Figure S4 B), and *NLRP3* transcripts decreased in the PLX3397 group compared with the CD group (Figure S4 A). Moreover, the expression of inflammation‐related molecules was measured in the striatum. It was revealed that after MPTP injection, *Iba1* and *IL1α* transcripts increased at 1 day, and *GFAP* increased at 1 day and 3 days, in both the CD and PLX3397 groups. The expression levels of *Iba1*[F (4, 29) = 4.089, *P* = .0095]*, C1q*, *IL1α* and *IL6* were markedly reduced in microglial‐depleted mice, whereas *GFAP*, *TNFα*, *IL1β*, *COX2* and *iNOS* transcripts did not differ between the CD and PLX3397 groups (Figure 4C).

**FIGURE 5:**
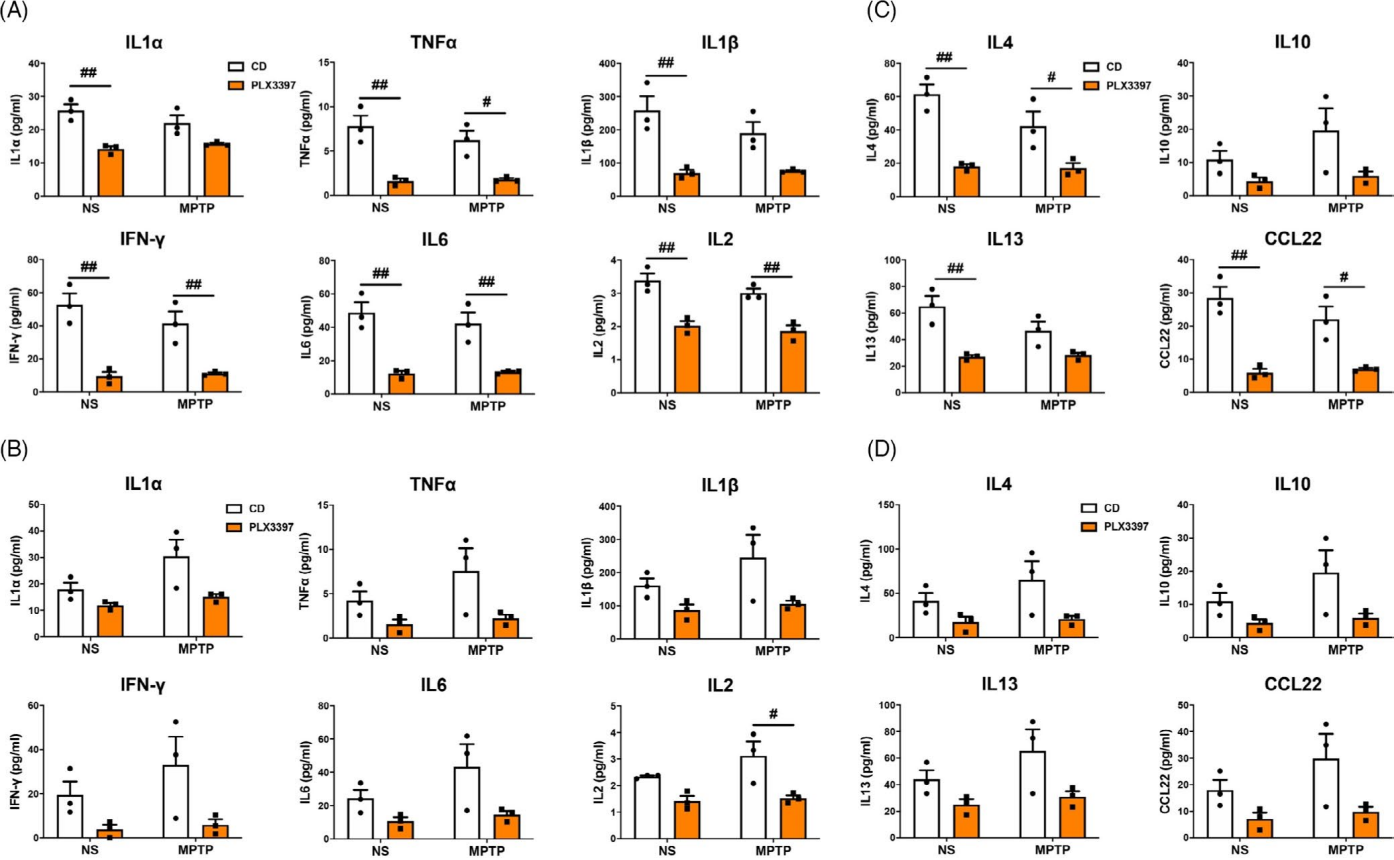
Protein levels of pro‐inflammatory and anti‐inflammatory cytokines in the nigrostriatal pathway at 1 day after MPTP administration. (A) The expression of pro‐inflammatory cytokines IL1α, TNFα, IL1β, IFN‐γ, IL6 and IL2 in the SN. (B) The expression of pro‐inflammatory cytokines IL1α, TNF‐α, IL1β, IFN‐γ, IL6 and IL2 in the striatum. (C) The expression of anti‐inflammatory cytokines IL4, IL10, IL13 and CCL22 in the SN. (D) The expression of anti‐inflammatory cytokines IL4, IL10, IL13 and CCL22 in the striatum. NS: normal saline; CD: control diet; PLX3397: PLX3397‐formulated diet. Two‐way ANOVA followed by Holm‐Sidak’s multiple comparisons test was used for statistical analysis. ^#^ *P* < .05, ^##^ *P* < .01, CD groups vs PLX3397 groups. n = 3

### 3.5 Partial depletion of microglia mitigates the production of inflammatory mediators in the nigrostriatal pathway

To evaluate whether microglial depletion affects the production of inflammation‐related mediators in the nigrostriatal pathway after MPTP exposure, we detected both the pro‐inflammatory and the anti‐inflammatory cytokines were detected by Multiplex immunoassay. Pro‐inflammatory cytokines, including IL1α, TNFα, IL1β, IFN‐γ, IL6 and IL2, were significantly decreased in the substantia nigra of PLX3397‐NS mice compared with CD‐NS mice; 1 day after MPTP treatment, the protein levels of TNFα, IFN‐γ, IL6 and IL2 were markedly reduced, whereas IL1α and IL1β proteins showed no difference between the CD and PLX3397 groups. Such changes did not occur in the striatum, where only IL2 was decreased in PLX3397‐MPTP mice at 1 day post‐injection compared with CD‐MPTP mice, and the other five cytokines (IL1α, TNFα, IL1β, IFN‐γ and IL6) did not alter (Figure 5A, B). Anti‐inflammatory cytokines IL4 and CCL22 were significantly reduced in the SN, but not in the striatum of PLX3397‐treated mice compared with CD‐treated mice in both NS and MPTP groups, and IL13 was dramatically reduced in the SN, but not in the striatum of PLX3397‐NS mice, while IL10 levels did not change in the nigrostriatal pathway (Figure 5C, D).

**FIGURE 6:**
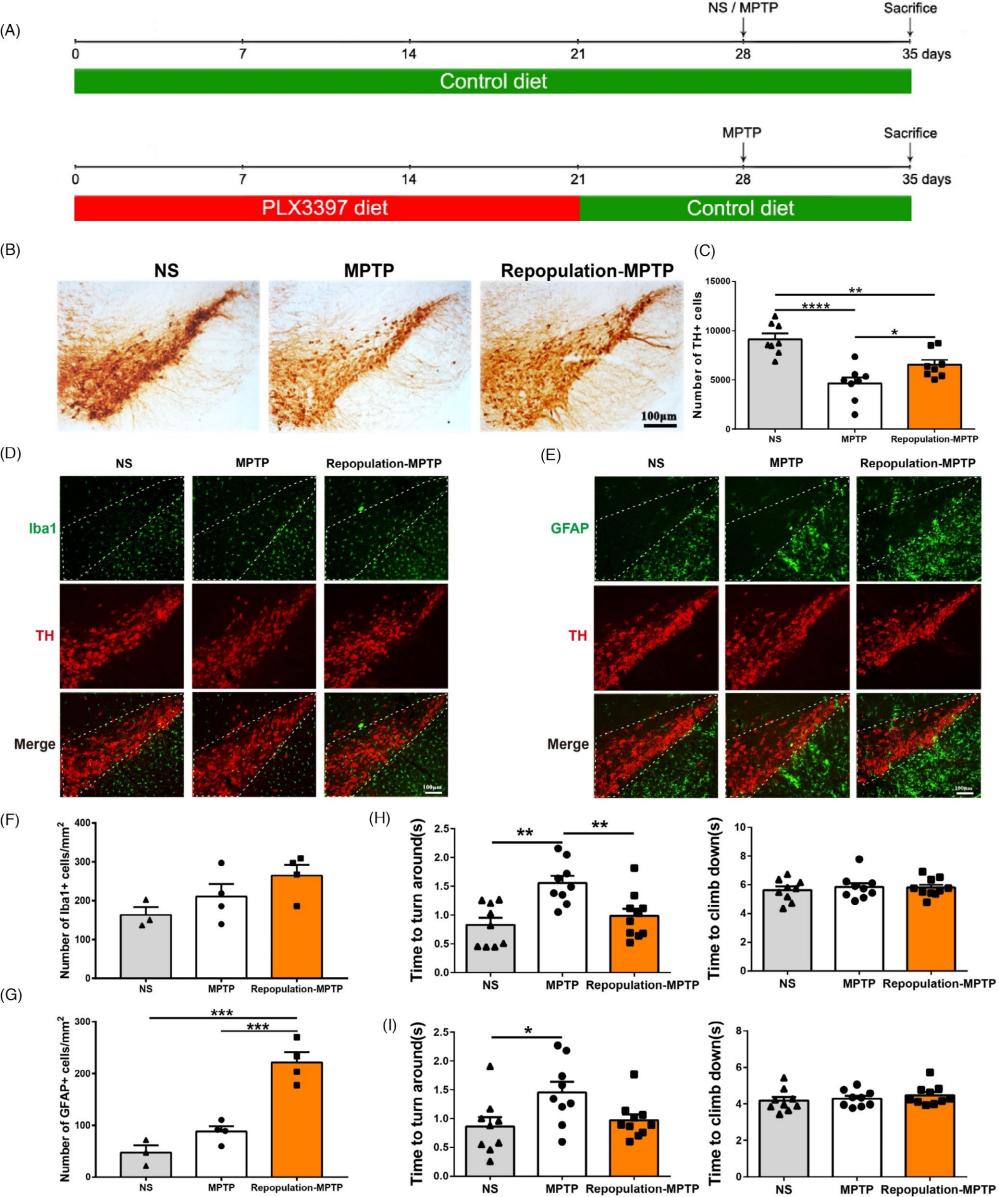
The effect of repopulated microglial cells in the substantia nigra of mice at 7 days after MPTP administration and the results of the Pole test. (A) Schematic schedule of the MPTP‐driven PD mice models with repopulated microglia. (B, C) Immunohistochemical staining (B) and stereological count showing TH^+^ cells (C) in the SNpc. NS group: CD‐NS‐CD; MPTP group: CD‐MPTP‐CD; repopulation‐MPTP group: PLX3397/21d‐CD/7d‐MPTP‐CD. n = 8. (D, F) Immunofluorescence staining of Iba1^+^ (green) and TH^+^ (red) in the SNpc; Scale bar: 100 μm (D) and quantification of Iba1^+^ microglial cells in the SNpc (F). n = 3‐4. (E, G) Immunofluorescence staining of GFAP^+^ (green) and TH^+^ (red) in the SNpc; Scale bar: 100 μm (E) and quantification of GFAP^+^ astrocytic cells in the SNpc (G). n= 3‐4. (H, I) The results of the Pole test at 3 days (H) and 7 days (I) after MPTP administration; n = 9‐10. NS: normal saline; CD: control diet; PLX3397: PLX3397‐formulated diet. One‐way ANOVA followed by Holm‐Sidak’s multiple comparisons test was used for statistical analysis. ^*^ *P* < .05, ^**^ *P* < .01, ^****^ *P* < .0001

### 3.6 Fully repopulated microglia lead to neuroprotection in MPTP‐induced PD mice

Microglia can restore rapidly after the withdrawal of PLX3397. Here, the effects of repopulated microglia in the mouse model of PD were investigated. Two paradigms of repopulation were adopted (Figure S5A, Figure 6A). First, repopulation of microglia by the cessation of PLX3397 at the time of MPTP administration yielded no difference in the nigrostriatal degeneration compared to the mice fed with a control diet and PLX3397‐formulated diet during the whole experimental process, at 7 days post‐MPTP injection (Figure S5B, C). In the second paradigm, after the full recovery of microglia in the mouse brain, MPTP was injected to induce the PD model. We found that at 7 days post‐MPTP injection, fully repopulated microglia brought about the mitigated reduction in dopaminergic neurons in mice compared with CD‐treated mice (Figure 6B, C). However, the numbers of microglia did not differ (Figure 6D, F). Astrocyte number was markedly increased only in the SN of MPTP‐intoxicated mice with replenished microglia (Figure 6E,G). Further, MPTP‐treated mice exhibited apparent motor deficits in the Pole test at 3 and 7 days post‐injection, whereas MPTP‐intoxicated mice with replenished microglia required a shorter time to turn around; no apparent difference was detected in terms of the time to climb down (Figure 6H,I).

### 3.7 Fully repopulated microglia confer to the elevated expression of neurotrophic factors, phagocytosis‐related molecules, but not the inflammation‐related molecules

**FIGURE 7:**
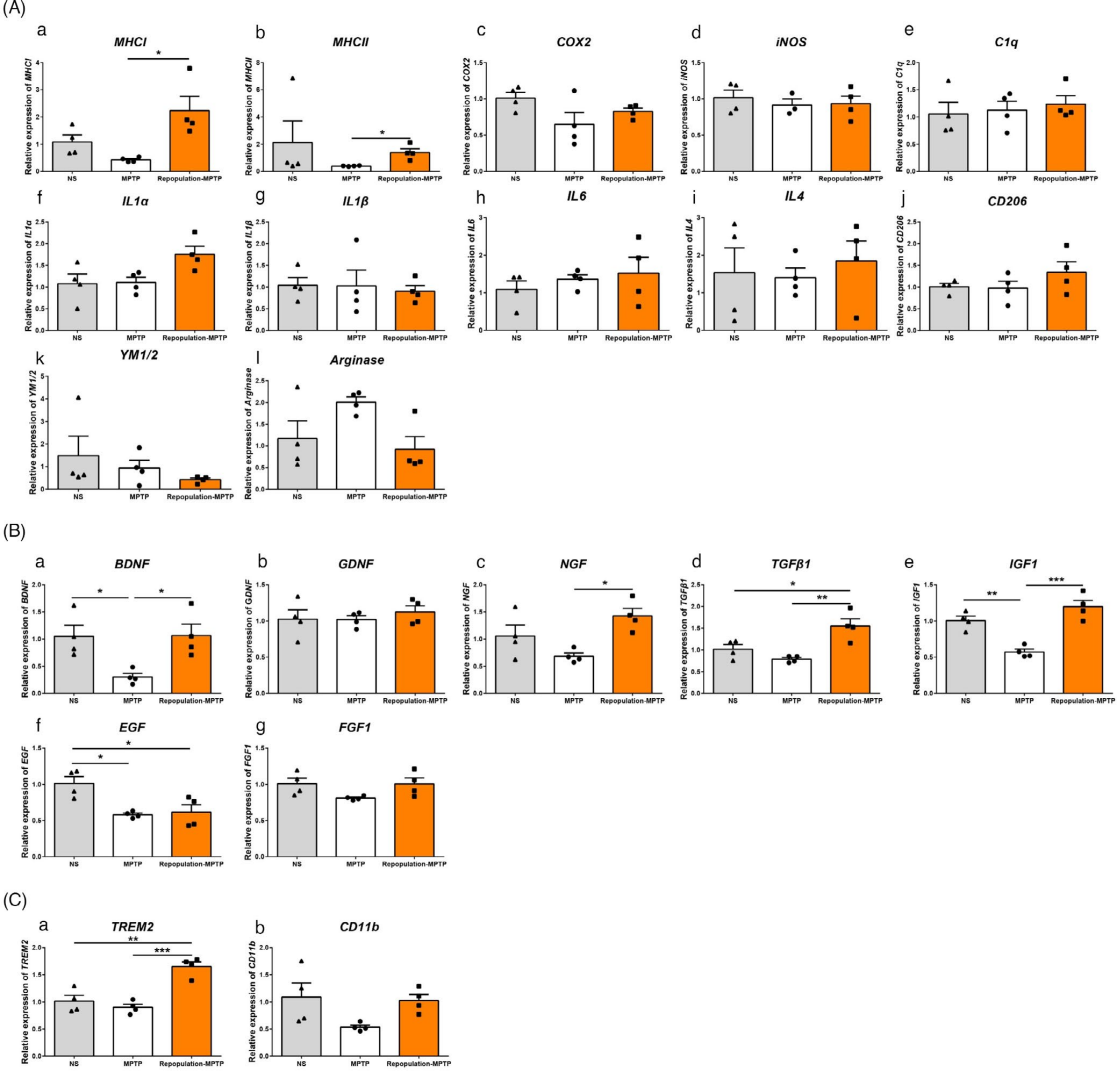
The transcripts of inflammation‐related molecules (A), neurotrophic factors and phagocytosis‐related molecules in mice with repopulated microglia at 7 days after MPTP administration. (A) The transcripts of *MHCI* (a), *MHCⅡ*(b), *COX2* (c), *iNOS* (d), *C1q* (e), *IL1α* (f), *IL1β* (g), *IL6* (h), *IL4* (i), *CD206* (j) *YM1/2* (k) and *Arginase* (l) in the striatum. (B) The transcripts of *BDNF* (a), *GDNF* (b)*, NGF* (c), *TGFβ1* (d), *IGF1* (e), *EGF* (f), and *FGF1* (g) in the striatum. (C) The transcripts of *TREM2* (a) and *CD11b* (b) in the striatum. *GAPDH* served as the reference gene. NS group: CD‐NS‐CD; MPTP group: CD‐MPTP‐CD; repopulation‐MPTP group: PLX3397/21d‐CD/7d‐MPTP‐CD. NS: normal saline; CD: control diet; PLX3397: PLX3397‐formulated diet. One‐way ANOVA followed by Holm‐Sidak’s or Dunn’s (as in A‐b) multiple comparisons test was used for statistical analysis. *P < .05, **P < .01, ***P < .001. n = 4

To gain insight into the neuroprotective function of repopulated microglia, we investigated the expression of neurotrophic factors, phagocytosis‐ and inflammation‐related molecules qPCR at 7 days post‐injection. First, MPTP treatment upregulated *Iba1* transcripts in mice of both CD and repopulation groups (Figure S6A), and increased the transcripts of *GFAP*, *major histocompatibility complex class Ⅰ* (*MHC Ⅰ*) and *class Ⅱ* (*MHC Ⅱ*) only in mice with repopulated microglia (Figure S6B, Figure 7A). Though MHC Ⅰ & Ⅱ are closely related to inflammatory reaction in MPTP‐driven PD mice,^26^ neither the pro‐inflammatory molecules (including *COX2, iNOS, C1q, IL1α, IL1β* and *IL6*) nor the anti‐inflammatory molecules (*IL4*, *CD206*, *YM1/2* and *Arginase*) showed expressing alternation in mice of both CD and repopulation groups after MPTP administration (Figure 7A). Second, the transcripts of neurotrophic factors were examined. Neurotrophic factors, such as brain‐derived neurotrophic factor (BDNF), glial cell‐derived neurotrophic factor (GDNF), nerve growth factor (NGF), transforming growth factor β (TGFβ), insulin‐like growth factor 1 (IGF1), epidermal growth factor (EGF) and fibroblast growth factor 1 (FGF1), are involved in neuronal survival, maintenance and regeneration, and glial activation, malfunctions of which contribute to the pathogenesis of PD.^34^, ^35^, ^36^, ^37^, ^38^, ^39^, ^40^ Our results showed that the striatal transcripts of *BDNF* and *IGF1* decreased sharply in the CD group after MPTP treatment compared with the NS group and MPTP‐treated repopulation group (Figure 7B). *NGF* transcript was markedly higher in mice with repopulated microglia compared to the CD group after MPTP injection (Figure 7B). *TGFβ* transcript exhibited robust upregulation in MPTP‐treated mice of repopulation group compared with the other two groups (Figure 7B), while EGF transcripts decreased both in the CD and repopulation groups after MPTP administration (Figure 7B). The transcripts of *GDNF* and *FGF1* did not change (Figure 7B).

Finally, since glial cells can phagocyte apoptotic cells, therefore avoiding further inflammation,^41^, ^42^ we measured the transcripts of phagocytosis‐related molecules, including *triggering receptor expressed on myeloid cells 2* (*TREM2*) and *complement receptor 3* (*CD11b*). The transcript level of *TREM2*, but not *CD11b* increased dramatically in microglial repopulation groups (Figure 7C).

## 4 DISCUSSION

Protractive neuroinflammation featured by the elevation of pro‐inflammatory cytokines and over‐activation of glial cells is a common characteristic in the brains of PD patients and PD animals. Accumulating evidence suggests that microglial activation is associated with Parkinson's disease^43^, ^44^, ^45^ ; however, the role of microglia in PD is still ambiguous. Microglia can be neuroprotective or neurotoxic, leading to beneficial or detrimental effects on the disease progression of PD.^46^, ^47^, ^48^ Under physiological conditions, microglia act as sensors and are neuroprotective. On the one hand, activated microglial cells upregulate the expression of pro‐inflammatory enzymes (such as COX2, iNOS), cytokines (IL1β, IL6, TNF‐α, etc) and chemokines, and release neurotoxic mediators such as NO and ROS. On the other hand, they also show increased expression of anti‐inflammatory cytokines and neurotrophic factors.^49^, ^50^, ^51^, ^52^ Thus, identifying effective ways to control and resolve chronic neuroinflammation is a pressing issue in PD.

Microglial depletion is a powerful approach to decipher the roles of microglia in various brain injuries. Beneficial, detrimental, dual roles and even null impacts of microglia in differential disease processes have been reported. Microglial ablation exacerbates post‐ischaemic inflammation and brain injury,^20^ and aggravates the severity of acute and chronic seizures in mice,^53^ suggesting the neuroprotective effects of microglia in such brain injury models. However, the elimination of microglia mitigates brain injury after intracerebral haemorrhage,^54^ improves cognition in transgenic Alzheimer's disease (AD) mice,^55^, ^56^ increases perineuronal nets and reduces disease‐related characteristics in Huntington's disease mice.^57^ A reduction in microglia during the early stage of ataxia disease—spinocerebellar ataxia type 1(SCA1)—ameliorates the motor deficits in SCA1 mice.^58^ Recently, Guo et al reported that microglial depletion partially suppresses α‐syn aggregation and transmission in the brain.^59^ All the evidence implies a deleterious role of microglia in brain diseases. Moreover, microglia exert beneficial effects during diphtheria toxin‐induced hippocampal neuronal lesions and, meanwhile, impede the recovery process,^19^ indicating a dual role of microglia. In addition, microglia might play a redundant role in specific brain insults. Hilla et al reported that microglia neither promoted nor inhibited neuronal degeneration or axonal regeneration after acute visual system injury.^60^ Streibel et al also found that microglia are not required for prion infection‐induced retinal photoreceptor degeneration.^61^ Moreover, the absence of microglia does not affect tau pathology in hTau mice.^62^ Therefore, the relationship between microglial and neurological disorders is quite complicated, depended on the disease stages and the diseases per se.

Microglia rely on the colony‐stimulating factor 1 receptor (CSF1R) signalling to proliferate and survive.^14^ CSF1R inhibition, using a selective pharmacological ATP competitive inhibitor GW2580, does not deplete microglia but attenuates disease‐stimulated microglial proliferation. GW2580 manifests protective effects in multiple brain disorders, including AD,^63^ amyotrophic lateral sclerosis,^64^ spinal cord injury,^65^ and PD as well.^66^ Notably, in rodent models of PD, the controversial effects of microglial depletion were brought up. PLX3397, a pharmacological receptor tyrosine kinase inhibitor of CSF1R, is widely used to eliminate microglial cells. However, controversial effects of microglia have been reported in MPTP‐, 6‐OHDA‐ or rotenone‐induced PD models.^18^, ^22^, ^23^, ^24^ To clarify the function of microglia in Parkinson's disease, in this study, we fed mice with a PLX3397‐containing diet for different days to test the efficiency of microglial depletion in the nigrostriatal pathway. Our data reveal that 21‐day treatment with PLX3397 dramatically reduced the microglial population in the substantia nigra, and had no noticeable effect on the nigrostriatal dopaminergic pathway in mice. Notably, the numbers of dopaminergic neurons and astrocytes showed no significant differences between the PLX3397‐fed and the control diet‐fed mice; the striatal neurotransmitters and mouse behaviours were not affected by microglial depletion as well. We used both quantitative PCR and Multiplex immunoassay to assess the expression of inflammation‐related factors. Among pro‐inflammatory cytokines TNFα, IL1α, IL1β, IFN‐γ and IL6, only IL6 showed nearly consistently changes at both transcription and protein levels. The discrepancy might originate from the methods per se. Moreover, the transcripts of inflammasome complex genes including *NLRP3, ASC, Caspase1, IL1β* and *IL‐18* exhibited different patterns; notably, the expression of *IL‐18* was upregulated in CD‐MPTP mice at the later time point of 14 days post‐injection. In general, the depletion of microglia mitigated the cerebral inflammation‐related gene expression in mice. Both pro‐inflammatory and anti‐inflammatory cytokines were reduced in the nigrostriatal pathway of PLX3397‐MPTP mice after the treatment. Our results imply that microglial depletion in mice can attenuate the inflammatory response induced by MPTP. We noted that the complement gene C1q was significantly downregulated in the nigrostriatal pathway of PLX3397‐fed mice, which agrees that microglia is the dominant source of C1q in mouse brain.^67^ C1q plays a critical role in the innate immune system. Fraser et al reported a protective role for C1q in the CNS during the early stages of cell death by enhancing microglial clearance of apoptotic cells and suppressing pro‐inflammatory cytokines.^41^ In our study, at 1 day post‐injection, dopaminergic neurons and microglial cells did not change in the SNpc of CD‐MPTP mice; however, dopaminergic neurons in PLX3397‐MPTP mice showed a significant reduction compared with CD‐NS mice. Downregulation of C1q in the nigrostriatal pathway of microglial‐depleted mice might contribute to the early demise of dopaminergic neurons. These results suggest a protective role of microglia at this very early stage of PD. Hereafter, in the SNpc, the microglial numbers in CD‐MPTP mice reached the climax at 3‐7 days post‐injection, whereas the numbers maintained relatively constant in PLX3397‐MPTP mice. Moreover, in the striatum, microglial cells in CD‐MPTP mice increased at 1 day and were restored at 5 days post‐injection and they did not alter in PLX3397‐MPTP mice. Thus, PLX3397 could inhibit microglial proliferation in both the SNpc and the striatum of mice after the treatment of MPTP. Partial microglial depletion rarely impacted the process of Parkinson's disease, indicated by the levels of TH proteins in the substantia nigra and the striatum, striatal TH^+^ fibre density and neurotransmitters, and motor functions. Astrocytes play a delayed effect by amplifying and sustaining inflammation. In this study, astrocytes exhibited no difference in microglial‐depleted mice and normal mice with or without MPTP treatment. The discrepancy between Yang's, Oh's studies and ours might result from different rodent models, different neurotoxin dosages and uneven microglial depletion efficiency. Nevertheless, we could not rule out the possibility that the residual microglia might completely fulfil the functions of microglia. All in all, a relevant microglial residual response, and together with the astrocytosis might account for the lack of neuroprotection in PLX3397‐fed mice.

Previous studies and our data showed that the microglia rapidly repopulated the brain after PLX3397 withdrawal for 4‐7 days, which was reported to be stemmed from the residual microglia.^68^ Repopulated microglia appear to be less reactive, which could dampen lesion‐induced inflammation. Willis et al have found that repopulated microglia promote brain repair in a mouse model of traumatic brain injury.^69^ Replacement of microglia in the aged brain reverses cognitive deficits in mice.^70^ Rice et al have reported that removing the reactive microglia and repopulating with new microglial cells offer a strategy to resolve neuroinflammation and promote recovery.^71^ Microglial repopulation promotes an anti‐inflammatory, trophic microenvironment and normalizes pro‐inflammatory gene expression in organotypic hippocampal slice cultures.^72^ Additionally, Willis et al have reported that repopulated microglia support functional neurogenesis during a critical time window after traumatic brain injury in an IL6‐dependent manner.^69^ Microglia in the mammalian brain can be manipulated into a neuroprotective and regenerative phenotype to help repair and alleviate cognitive deficits caused by brain injury.^69^ Therefore, all of the above mentioned support the possibility of microglial depletion/repopulation as a strategy to reverse chronic neuroimmune activation. We found that the effects of repopulated microglia in the MPTP mouse model depended on the time windows of PLX3397 cessation. 7 days after the removal of PLX3397, mice with completely repopulated microglia were challenged with MPTP. In the case, complete microglial repopulation brought about dopaminergic protection and motor improvement. However, the dopaminergic neuronal loss did not differ between PLX3397‐fed mice with drug cessation at the same time of MPTP exposure and MPTP‐exposed mice fed with the control diet during the whole experimental process. Enhanced expression of neurotrophic factors and molecule(s) involved in phagocytosis may confer to the neuroprotection of repopulated microglia in MPTP‐challenged mice. Finally, since multiple MPTP mouse models are used world widely, including acute, subacute and chronic, the limitations of the acute MPTP mouse model are obvious, such as that dopaminergic neurons die quickly and little progression in the loss of dopaminergic system is observed.^73^, ^74^, ^75^ Other MPTP administration regimens are genuinely worthy of being applied in evaluating the effects of microglial depletion and replenishment on PD‐like damages.

In summary, microglial cells inhibit the initial damage of dopaminergic neurons in PD. Hereafter, Parkinson's disease progresses move forth in a way independent of microglial depletion in the MPTP‐induced mouse model. Microglial depletion/repopulation might be a potential approach for the intervention of PD.

## AUTHOR CONTRIBUTIONS

FH, JF, RS and RS proposed and supervised the study. FH, QL, CS and ZL wrote the manuscript. QL, CS, ZL, JW, HD, XZ, YM, ZW, L.C and MY performed the experiments. All authors contributed to the interpretation of data and the revision of the manuscript.

## Supporting information

Fig S1‐S6

## Data Availability

The data that support the findings of this study are available from the corresponding author upon reasonable request.
