# Supplementary material for: Partial depletion and repopulation of microglia have different effects in the acute MPTP mouse model of Parkinson’s disease
Source: Cell Prolif. 2021 Jul 26;54(8):e13094. doi: 10.1111/cpr.13094 (PMC8349650; doi:10.1111/cpr.13094)
Supplement: Supplementary file 1 — Fig S1‐S6 [file CPR-54-e13094-s001.docx]

**Supplementary** **Materials**


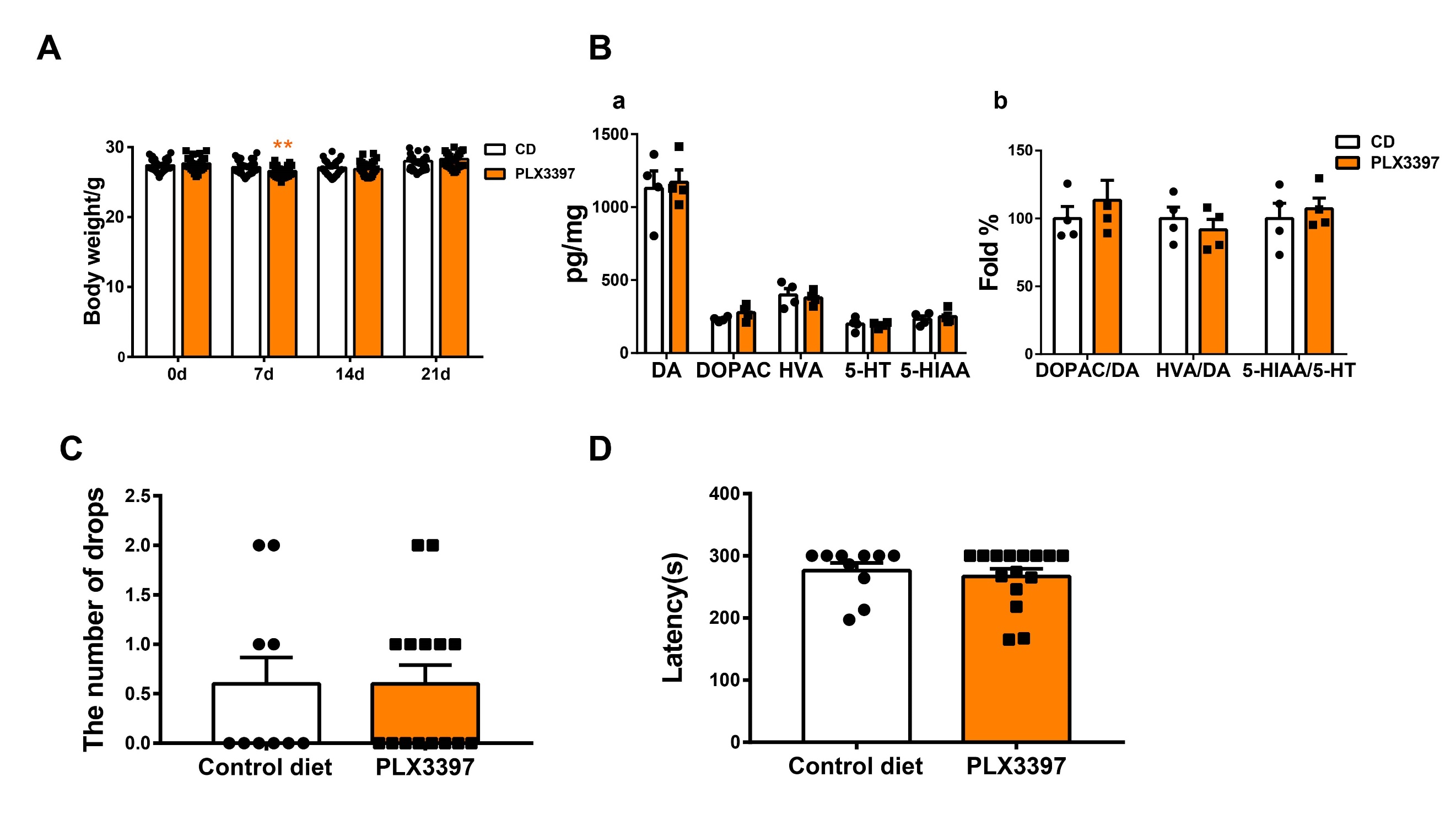


**Fig. S1 The effect of microglial depletion on the body weight, striatal neurotransmitters, and mouse performance in the Rotarod test. (A)** Mouse body weights of the CD and PLX3397 groups. Two-way ANOVA followed by Holm-Sidak’s multiple comparisons test was used for statistical analysis. ***p*<0.01, vs 0d PLX3397 group; n=30. **(B)** HPLC assays of the striatal dopamine and its metabolites DOPAC and HVA, 5-HT and its metabolite 5-HIAA after MPTP administration **(a)**. Ratios of DOPAC to DA, HVA to DA, and 5-HIAA to 5-HT **(b)** were also shown. Unpaired two-tailed Student’s t-test was used for statistical analysis. n=4. **(C, D)** The number of drops and latency to fall during the Rotarod test. Unpaired two-tailed Student’s *t*-test was used for statistical analysis. n=10-15. CD: control diet; PLX3397: PLX3397-formulated diet.


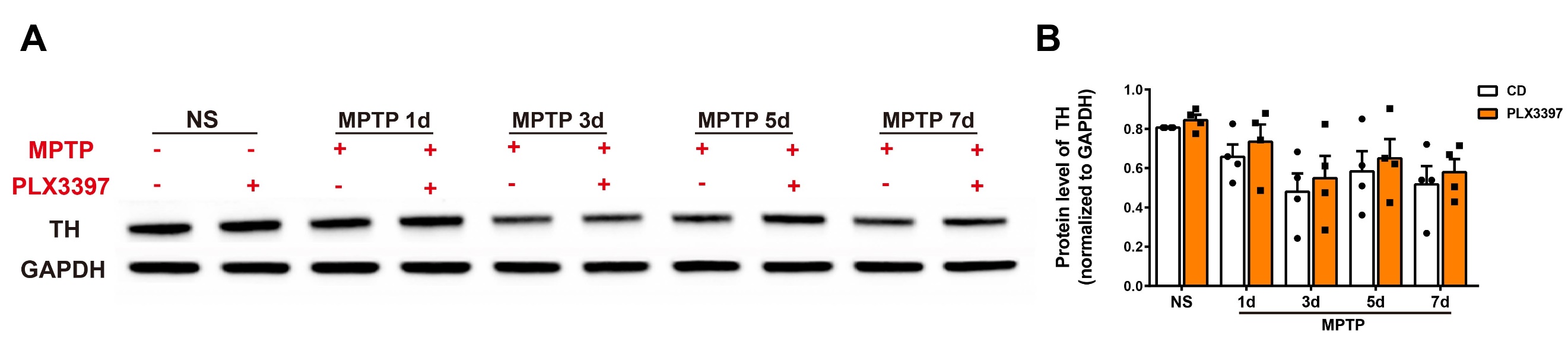


**Fig. S2 Analysis of TH protein expression in the substantia nigra of mice. (A)** Western blot analysis of TH protein expression, GAPDH served as the control. **(B)** Quantification of TH protein levels. NS: normal saline; CD: control diet; PLX3397: PLX3397-formulated diet. Two-way ANOVA followed by Holm-Sidak’s multiple comparisons test was used for statistical analysis. n=4.


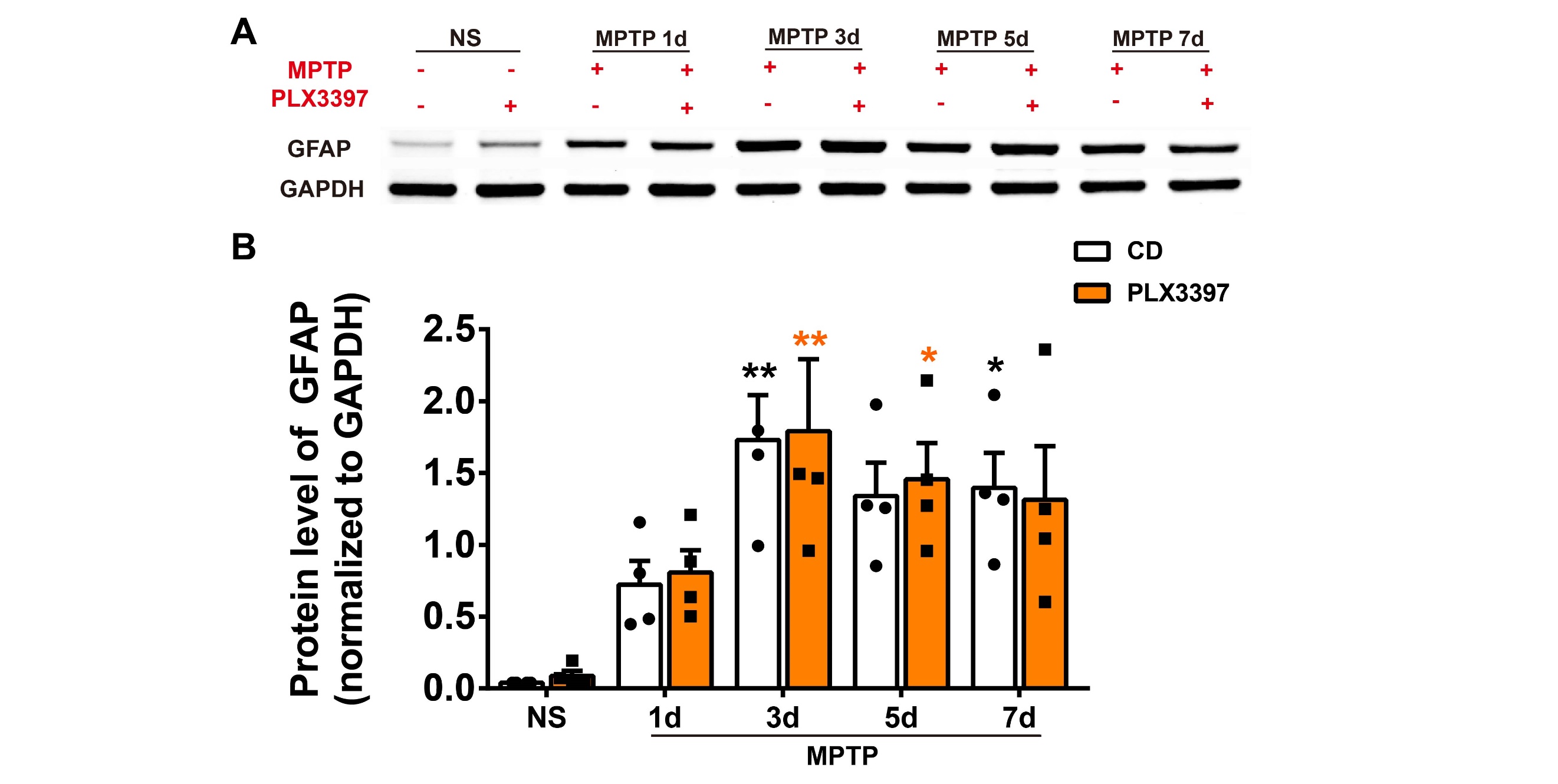


**Fig. S3 Analysis of the striatal protein levels of GFAP in mice. (A)** Western blot analysis of GFAP expression. GAPDH served as the control. **(B)** Quantification of GFAP protein levels. NS: normal saline; CD: control diet; PLX3397: PLX3397-formulated diet. Two-way ANOVA followed by Holm-Sidak’s multiple comparisons test was used for statistical analysis. **p*<0.05, ***p*<0.01, *vs* respective NS groups. n=4.


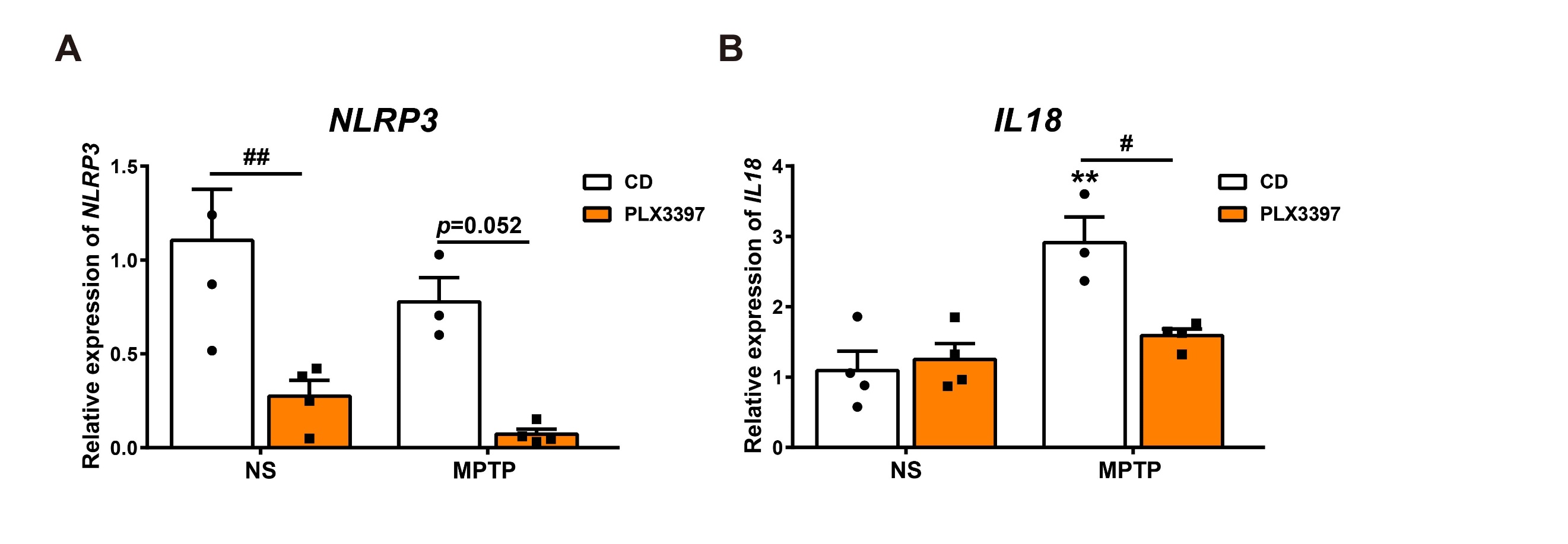


**Fig. S4 The transcripts of *NLRP3* and *IL18* in mice at 14 days after MPTP administration.** **(A)** The transcripts of *NLRP3* in the substantia nigra. **(B)** The transcripts of *IL18* in the substantia nigra. *GADPH* served as the reference gene. NS: normal saline; CD: control diet; PLX3397: PLX3397-formulated diet. Two-way ANOVA followed by Holm-Sidak’s multiple comparisons test was used for statistical analysis. ***p*<0.01, *vs* CD-NS group. #*p*<0.05, ##*p*<0.01, CD groups *vs* PLX3397 groups. n=3-4.


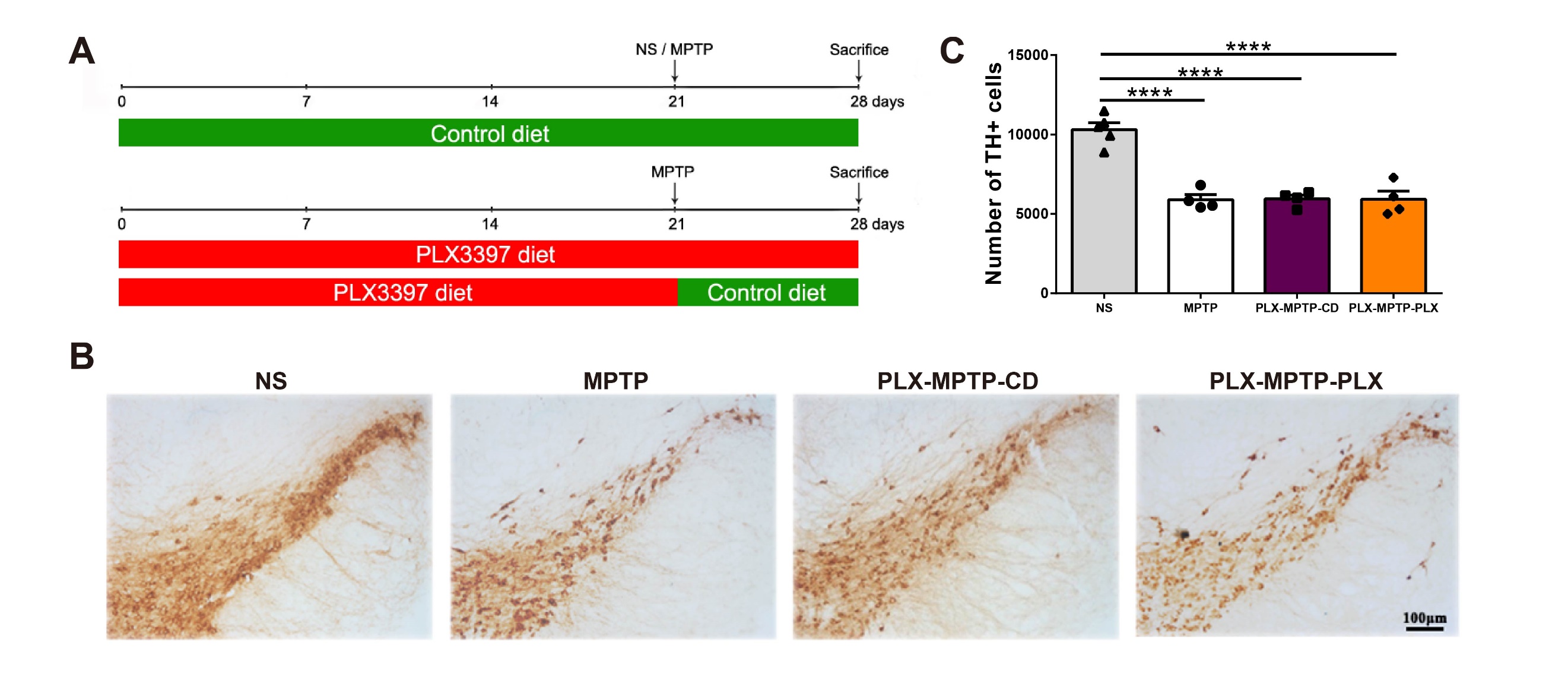


**Fig. S5 The effect of partial depletion and repopulation of microglial cells on dopaminergic neurons in the substantia nigra of mice at 7 days after MPTP administration.** **(A)** Schematic illustration of the MPTP-driven PD mice models. **(B)** Immunohistochemical staining showing TH^+^ cells in the SNpc. Scale bar: 100 mm. **(C)** The stereological count of TH^+^ cells in the SNPc. NS group: CD-NS-CD; MPTP group: CD-MPTP-CD; PLX-MPTP-CD group: PLX3397/21d-MPTP-CD; PLX-MPTP-PLX group: PLX3397/21d-MPTP-PLX3397. NS: normal saline; CD: control diet; PLX, PLX3397: PLX3397-formulated diet. One-way ANOVA followed by Holm-Sidak’s multiple comparisons test was used for statistical analysis. *****p*<0.0001. n=4-5.


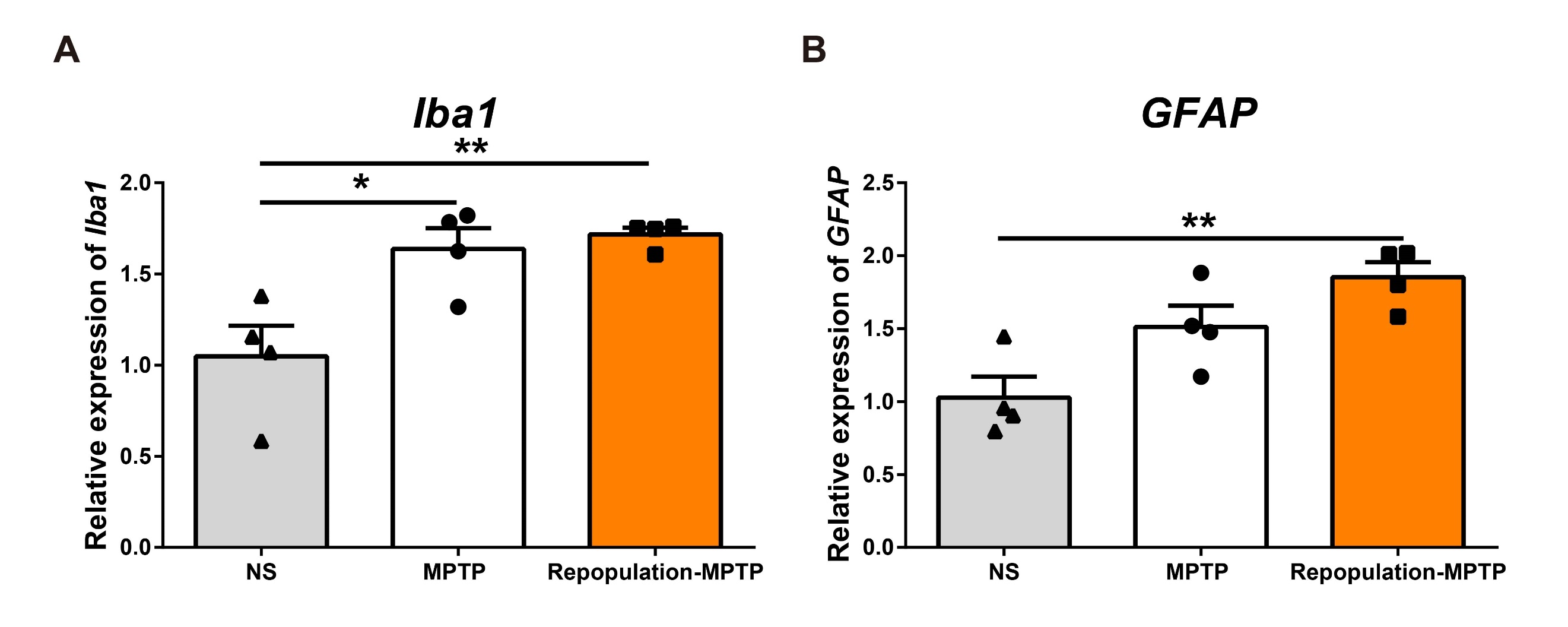


**Fig. S6 The transcripts of *Iba1* and *GFAP* in mice at 7 days after MPTP administration. (A)** The transcripts of *Iba1* in the striatum. **(B)** The transcripts of *GFAP* in the striatum. *GAPDH* served as the reference gene. NS group: CD-NS-CD; MPTP group: CD-MPTP-CD; Repopulation-MPTP group: PLX3397/21d-CD/7d-MPTP-CD. NS: normal saline; CD: control diet; PLX3397: PLX3397-formulated diet. One-way ANOVA followed by Holm-Sidak’s multiple comparisons test was used for statistical analysis. **p*<0.05, ***p*<0.01. n=4.
